# XAI-MedNet: A Next-Generation Explainable AI Framework for Contrast-Enhanced Skin Lesion Classification via Entropy-Controlled Optimization

**DOI:** 10.3390/bioengineering13050506

**Published:** 2026-04-27

**Authors:** Abdulrahman Alabduljabbar, Tallha Akram, Youssef N. Altherwy, Muhammad Adeel Akram, Imran Ashraf

**Affiliations:** 1 Department of Information Systems, College of Computer Engineering and Sciences, Prince Sattam Bin Abdulaziz University, Al-Kharj 11942, Saudi Arabia; a.alabduljabbar@psau.edu.sa (A.A.);; 2 Department of Computer Engineering, COMSATS University Islamabad, Wah Campus, Wah Cantt 47040, Pakistan; 3 Computer Engineering Lab, QCE Department, EEMCS, TU Delft, 2628 CD Delft, The Netherlands

**Keywords:** Explainable AI, interpretable machine learning, skin lesion classification, BAT optimization, artificial bee colony optimization, whale optimization, feature selection, CNN models, evolutionary techniques

## Abstract

Explainable Artificial Intelligence (XAI) has become a critical requirement in medical image analysis, where transparency and interpretability are essential for clinical trust and decision support. Melanoma is recognized as one of the most deadly types of skin cancer, with its occurrence exhibiting an increasing pattern in recent times. However, detecting this cancer in its initial stages greatly increases patients’ chances of long-term survival. Various computer-based techniques have recently been proposed to diagnose skin lesions at their early stages. Even though the machine learning community has achieved a certain degree of success, there is still an unresolved research challenge regarding high error margins and the limited interpretability of automated systems. This study focuses on addressing both segmentation and classification tasks, with particular emphasis on two key concepts: (1) improving image quality to maximize distinguishability between foreground and background regions, thereby enhancing visual interpretability and segmentation accuracy and (2) eliminating redundant and cluttered feature information to generate the most discriminative and compact feature representations. The input images are initially processed using a novel metaheuristic contrast-stretching method to estimate image-specific key parameters, thereby enhancing lesion boundary clarity in a clinically interpretable manner. Following this, the improved images are fed into selected pre-trained deep models, including DenseNet-201, Inception-ResNet v2, and NASNet-Mobile. The extracted features from all pre-trained models are fused to produce resultant vectors, which are then refined using a bio-inspired feature selection method, termed entropy-controlled whale optimization, to retain only the most informative attributes. The selected discriminative feature set is subsequently classified using multiple classifiers. The results indicate that the proposed framework achieves superior performance compared to existing methods in terms of accuracy, sensitivity, specificity, and F1-score. Additionally, it facilitates a more explainable, transparent, and structured diagnostic pipeline appropriate for medical applications.

## 1. Introduction

Skin cancer is a prevalent type of cancer that affects a large number of people globally, with around 5.4 million cases identified each year. The escalating incidence of skin cancer in recent decades has positioned this ailment as a matter of particular concern within the realm of public health [1]. Accurate and prompt classification of skin cancer is crucial for determining the most effective treatment plans. The standard criteria for categorizing skin cancers include the disease’s origin, development pattern, and level of cellular differentiation [2].

Skin cancer presents in a variety of forms, including melanoma, squamous cell carcinoma, and basal cell carcinoma [3]. To achieve the best possible results when treating patients with any of these skin cancers, it is imperative that they be accurately classified into one of these three categories [4]. This is because different subtypes and stages of skin cancer require different treatment modalities. The process of determining which of these three categories a patient’s cancer falls into involves identifying the tumor’s individual characteristics, which then help the clinician make appropriate treatment decisions for that patient. As a result of advancements in computer vision (CV) and artificial intelligence (AI), healthcare practitioners have developed automated methods for classifying skin lesions. These systems support healthcare practitioners with accurate, timely diagnostic information for patients with suspected skin cancer [5]. When diagnosing a patient with suspected skin cancer, an automated classification system uses machine learning to evaluate and classify the dermal lesion based on its specific features and provides the clinician with potential treatment options based on the classification results. The implementation of automated classification systems has the potential to increase the accuracy and effectiveness of skin cancer diagnosis, thereby improving patient outcomes [6,7].

Several methodologies are currently used to identify skin cancer, ranging from visual examinations by medical professionals to advanced imaging techniques [8,9]:

- Visual inspection: Healthcare professionals assess suspicious moles or lesions using the naked eye or a dermatoscope, a handheld device that magnifies the skin.
- Biopsy: This procedure involves extracting a small portion of skin tissue for microscopic analysis, typically performed when an abnormal lesion is detected during a visual examination.
- Dermoscopy: A non-invasive technique using a dermatoscope to reveal internal skin structures through magnification and polarized light.
- Reflectance confocal microscopy (RCM): A non-invasive method that captures high-resolution images of the dermis’ internal architecture using a laser.
- Computer-aided diagnosis (CAD): This approach utilizes machine learning algorithms to analyze images and label them as benign or malignant based on their features.
- Beyond improving predictive performance, modern CAD systems must be interpretable and transparent to achieve clinical acceptance. This study addresses these requirements by situating the proposed framework within Explainable AI, using interpretable preprocessing and entropy-driven feature selection.

**Figure 1:**
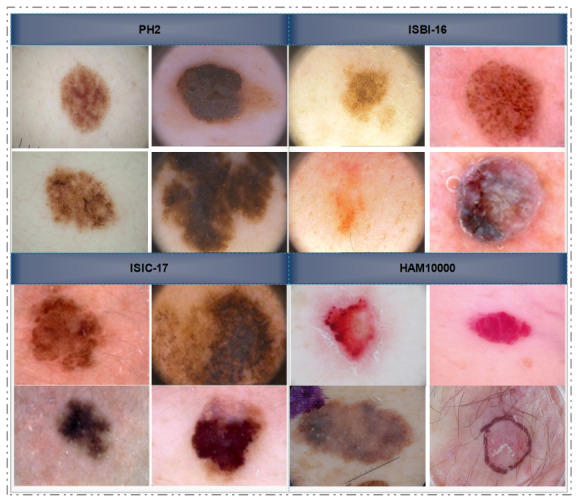
Comparative visualization of skin lesion diversity across benchmark dermoscopy datasets: (**Top-Left**) PH^2^, (**Top-Right**) ISBI-2016, (**Bottom-Left**) ISIC-2017, and (**Bottom-Right**) HAM10000.

In this work, we focus on both segmentation and classification tasks, emphasizing two core concepts. First, improving image quality allows for better distinction between the foreground and background regions; second, removing noisy and redundant information leads to more discriminative feature representations. Our key contributions include proposing a hybrid meta-heuristic model based on an extended BA-ABC for contrast stretching and introducing entropy-controlled whale optimization for feature selection. We selected publicly available datasets to evaluate our framework; Figure 1 illustrates a few samples.

## 2. Literature Review

The detection of skin lesion borders and classes has been the subject of many models presented by researchers [10,11,12,13]. The past decade has seen the widespread adoption of convolutional neural networks (CNNs), which have enabled more accurate segmentation and classification results [14]. However, in practice, it’s fairly common to downsample images to avoid unnecessary iterations and parameter calculations, which could result in the loss of certain features. Similarly, preprocessors can improve segmentation and classification results—but at the expense of increased processing time. In the CV domain, however, there’s currently no universal model for removing noise and improving contrast. Researchers, however, have made important contributions by suggesting a plethora of techniques to address this issue. In [15,16], the authors utilized contrast enhancement as a preprocessor to differentiate the region of interest (RoI). Additionally, they created a saliency map using a single channel of the RGB color space to generate a binary image. Furthermore, they explored a bio-inspired evolutionary algorithm, particle swarm optimization (PSO), for efficient boundary estimation. Following the addition of a stage for feature extraction, they turned to a metaheuristic approach using a genetic algorithm (GA) to identify the most discriminant feature set, followed by a classifier to categorize the features into a selected number of classes. Similarly, Khan et al. [17] presented a deep learning strategy for evaluating skin lesions. The images were first subjected to a decorrelation formulation before being segmented using Mask R-CNN. DenseNet was fed the segmented images to extract features, and a least squares, entropy-controlled SVM algorithm was used to rank the features based on their relevance. The authors tested their approach on three selected benchmark datasets including ISBI-2016, ISIC–2017 and HAM10000, achieving average accuracies of 96.3%, 94.8%, and 88.5%, respectively.

The authors in [18] introduced a mutually-guided convolutional neural network (CNN) architecture for the classification of skin lesions. Initially, they divided the lesions into segments by employing a modified coarse segmentation network. Subsequently, they employed a mask-guided CNN for classification. They tested their framework on two publicly available datasets, including ISIC2017 and PH^2^, achieving accuracies of 93.8% and 97.7%, respectively. Even with an imbalanced dataset, this approach has been shown to be helpful in segmenting and classifying skin lesions.

Similarly, Al-Masni et al. [19] proposed a unified deep learning model for segmenting and classifying a wide variety of skin lesions. For segmentation, they utilized four pre-trained deep CNN classification models, and for lesion extraction, they employed a full-resolution deep learning approach. Three skin datasets (ISBI-2016 and ISIC-2017, and ISIC-2018) were used to evaluate the proposed technique, and the findings were highlighted once the datasets were normalized correctly. Miglani and Bhatia [20] examined the efficacy of deep CNN models in identifying skin lesions. ResNet-50 and EfficientNet-B0 were used to implement transfer learning, with optimal parameters set. EfficientNet-B0 surpassed ResNet-50 on the HAM1000 dataset, with macro and micro AUC values of 0.93 and 0.97, respectively.

A skin lesion classification framework based on data augmentation was proposed by Cano et al. [21], in which a NASNet deep network was trained directly on the expanded dataset. The classification model’s accuracy was verified using the ISIC dataset. Similarly, Aziz et al. [22] utilized an SVM classifier to classify lesions, utilizing a pre-trained AlexNet model for feature extraction. For both feature selection and fusion using hybrid whale optimization and canonical correlation, Afza et al. [23] (2022) developed a hybrid strategy for classifying skin lesions across several classes. By utilizing the HAM10000 and ISIC-2018 datasets with the extreme learning machine (ELM) classifier, they were able to classify skin lesions with accuracies of 93.40% and 94.36%, respectively.

Recent research has increasingly focused on Vision Transformer (ViT)-based and hybrid methods to overcome the limitations of traditional convolutional architectures in skin lesion classification. An example of this is displayed in Aruk et al.’s study [24] where a hybrid architecture of ConvNeXt-Transformer was produced that uses CNN blocks for the extraction of localized textural features and ViT structures for modeling of long-distance spatial relationships. As cited by Aruk et al. [24], the model built from their original work achieved 94.30% accuracy on the HAM10000 Datastore. Furthermore, the GAMFuse system supplies another example of this type of work. At the beginning of this model, the Swin Transformer (ViT) was integrated, incorporating an attention mechanism to scale global information. Additionally, the GAMFuse system incorporated a Residual CNN to obtain local feature information at the most granular resolution [25]. Finally, GNNs were integrated into this architecture to model relationships between lesions on image-based datasets; the resultant model performance exceeded baseline measures that typify models trained under ’few-shot’ conditions. Collectively, these examples provide evidence that combining local and global feature representations yields state-of-the-art diagnostic performance on dermatological databases.

Despite the success of recent hybrid approaches, most existing methods primarily focus on architectural hybridization, such as combining CNNs with Transformer-based modules or integrating feature fusion strategies. These approaches typically enhance either feature extraction or classification performance but often overlook the importance of jointly optimizing multiple stages of the pipeline. In contrast, the proposed framework introduces a multi-stage hybrid design, where hybridization is applied at both the image preprocessing level (via entropy-guided contrast enhancement) and the feature selection level (via entropy-controlled whale optimization). This enables simultaneous improvement in visual interpretability and feature discriminability, distinguishing the proposed approach from existing hybrid models.

## 3. Problem Statement and Contributions

Recently, the importance of CAD systems in identifying and classifying skin lesions has increased significantly. Nevertheless, there are particular challenges that make skin lesion classification more complex. These factors include skin flakes, low contrast in lesion regions, air bubbles, hair, diffuse boundaries, and an imbalanced dataset. Furthermore, differences in rotation, lighting, and shearing of the same lesion across multiple images result from the skin’s flexibility, making the classification of those lesions even more challenging. Conventional feature selection methods exhibit reduced accuracy and incur high computational cost after the extraction phase. Therefore, hybrid meta-heuristic algorithms have been presented that demonstrated superiority in performing these functions. Unlike conventional hybrid frameworks that operate at a single stage, the proposed method introduces a dual-level hybridization strategy, integrating entropy-driven preprocessing and entropy-controlled feature selection to improve both interpretability and classification robustness.

The principal contributions of our research are:

- Proposal of a novel contrast stretching framework that combines an extended Bat algorithm with an Artificial bee colony (EBA-ABC) algorithm to improve the lesion visibility and boundary clarity.
- Proposal of a bio-inspired feature selection framework, entropy-controlled whale optimization algorithm, to address challenges related to the “*curse of dimensionality*” and over-fitting.
- Development of a transparent diagnostic workflow incorporating XAI methodologies to provide clinicians with traceable visual evidence, ensuring that model predictions for lesion malignancy are grounded in interpretable feature analysis.

Given a database of dermoscopic images, each image is required to be assigned a label of malignant or benign. For a dermoscopic image $I\subset R^{\left(h\times s\times l\right)}$ from the HSL color space, belonging to the given dataset $D^{\nu }$, where ν∈1,2,3,4 is the selected dataset. The set of images is $\left(I_{1}^{\nu }\right),\left(I_{2}^{\nu }\right),\ldots ,\left(IU^{\nu }\right)\subset D^{\nu }\in R^{\left(1\times \nu \right)}$. The dataset contains a set of U images, where each image is predetermined to have three channels. Additionally, the  corresponding label for each image is provided. With the proposed contrast stretching technique, each image is enhanced, followed by a color space transformation to the RGB space, $\Phi^{e}\in R^{R\times G\times B}$. The enhanced images are subsequently employed for transfer learning by utilizing pre-trained models to generate a set of feature vectors, ${\tilde{\Phi }}^{X}\in R^{\left(r\times c\right)}$. The features undergo a subsequent procedure known as feature selection, wherein the goal is to identify the most discriminative feature information while removing any redundant feature values, ${\tilde{\Phi }}^{fs}$.

The final representation of the cascaded system comprises a sequence of stages, including contrast stretching, feature fusion in conjunction with feature selection, and final labeling. The mathematical description of the proposed pipeline is given as:

(1) $${\tilde{\Phi }}^{le}≜\left(\Phi^{e},\;{\tilde{\Phi }}^{X},\;{\tilde{\Phi }}^{fs}\right)\in R^{\left(U\times 1\right)}$$

where ${\tilde{\Phi }}^{le}$ represents the class labels at the output of the hierarchical structural design.

## 4. Proposed Framework

**Figure 2:**
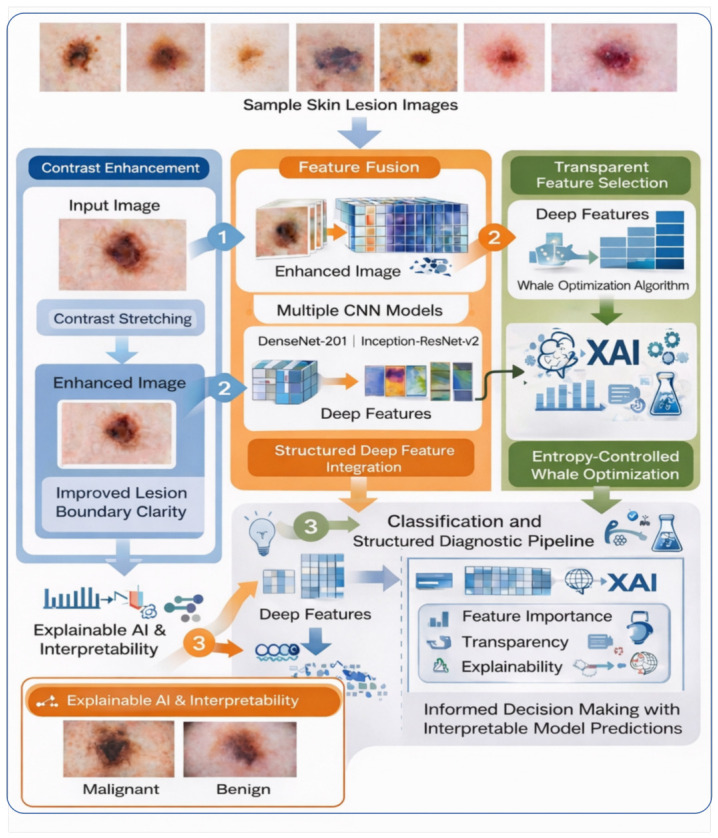
The proposed system architecture for interpretable skin lesion classification, integrating contrast enhancement, multi-CNN feature fusion, and entropy-controlled feature selection with Explainable AI (XAI).

In this section, we discuss the proposed framework depicted in Figure 2. The proposed architecture comprises several phases: image pre-processing, feature extraction after transfer learning, feature fusion in conjunction with selection, and classification, creating a semi-transparent diagnostic pipeline aligned with Explainable Artificial Intelligence (XAI) principles. Recall that we aim to tackle both segmentation and classification tasks, with a particular emphasis on two fundamental concepts: (1) An improved contrast stretching technique optimizes the framework’s ability to distinguish between the foreground and background regions, yielding visually interpretable outputs that provide image-level explainability, and (2) A robust feature selection technique mitigates the overfitting constraint while offering feature-level explainability by using a transparent, entropy-controlled mechanism to highlight the most discriminative attributes. Below, we discuss each of the steps outlined above.

### 4.1. Extended BA-ABC Algorithm

In this section, we discuss the image pre-processing technique, particularly the extended BA-ABC contrast stretching technique, that belongs to a class of nature-inspired algorithms. Nature-inspired algorithms are widely accepted for their ability to generate adaptive, innovative, and efficient solutions, especially those related to contrast enhancement and other image processing tasks [26,27]. Our proposed technique, the extended BA-ABC technique, is a hybrid model for boundary estimation based on bio-inspired bat and artificial bee colony algorithms. We refer the reader to the work published in [28,29] for a detailed review of the bat and artificial bee colony algorithms.

The proposed technique alternates between the BA and ABC algorithms to identify suboptimal solutions. This approach enhances the lightness channel $\psi^{L}$ of the RGB-transformed HSL image, resulting in a new enhanced lightness channel $\psi^{e}$. The contrast enhancement model preserves the original input image size, consisting of *M* rows and *N* columns. The transformation function of the proposed model is described in a generalized form as:

(2) $$\delta_{x,y}^{g}=T\left\{\;\delta_{x,y}^{f}\;\right\}\;\;\;\;\;\forall x\in M,\forall y\in N$$

The function depends on various statistical parameters, including the mean, standard deviation, and local-global attributes, which encompass edge pixels & intensity values, and the intensity distribution measurement, specifically the Gini coefficient. The transformation function consists of two components: the first component involves two parameters, Pβ and Pθ, and their combined impact with the local mean $\psi_{x,y}^{\mu }$ and the global mean $\mu^{g}$. The main advantage of the local mean $\psi_{x,y}^{\mu }$ is its dependence on the immediate neighborhood. The following portion of the transformation function improves smoothness and brightness conservation by utilizing the local mean $\psi_{x,y}^{\mu }$ and by incorporating the parameter $P_{\alpha }$ in the exponent.

(3) $$\delta_{x,y}^{g}=\left(P_{\delta }\frac{\mu^{g}}{\psi_{x,y}^{\sigma }+P_{\beta }}\right)\times \left[\delta_{x,y}^{f}-P_{\tau }\times \psi_{x,y}^{\mu }\right]+{\left[\psi_{x,y}^{\mu }\right]}^{P_{\alpha }}$$

Population generation in the bat algorithm is governed by the parameters Pα,Pβ,Pτ,Pδ. After optimization, these parameters are returned to the main function. The enhanced model is subsequently constructed using the improved cost function, defined as follows:

(4) $$C_{\psi^{e}}=\frac{log\left(log\left(\psi^{s}\right)\right)\times edgels\left(\psi^{e}\right)\times G\left(\psi^{e}\right)}{M\times N}$$

**Figure 3:**
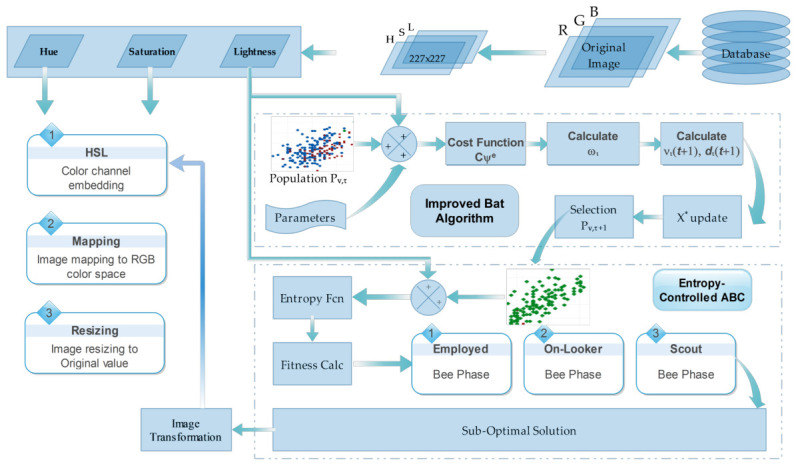
Procedural workflow of the proposed contrast enhancement technique, combining HSL color space transformation with Improved Bat and Entropy-Controlled ABC optimization.

The quality of the image is assessed based on a number of factors, including an increased level of edge pixels, high randomness in the distribution of pixels, and improved lightness values. The Sobel edge-detection filter identifies sharp changes based on both vertical and horizontal filters, along with the gradients. A log function is implemented twice in the cost function to subdue over-contrast stretching [27]. The Sobel edge detection filter is given as:

(5) $$\psi^{s}=\sum_{x\in M}\sum_{y\in N}\sqrt{(\frac{\partial \psi_{x,y}^{e}}{\partial_{v}}{)}^{2}+(\frac{\partial \psi_{x,y}^{e}}{\partial_{h}}{)}^{2}}$$

where ∂v and ∂h are the vertical and horizontal gradients. The Gini coefficient is added to the main cost function to measure the randomness or inequality in the distribution of pixel intensities. The Gini coefficient is calculated as:

(6) $$G\left(\psi^{e}\right)=\frac{1}{2{\left(M\times N\right)}^{2}\mu }\sum_{j=1}^{M}\sum_{k=1}^{N}\left|x_{j}-x_{k}\right|$$

where $x_{j}$ is the intensity of the $j^{th}$ pixel in the sorted list. The detailed flow diagram of the proposed extended contrast stretching model is given in Figure 3.

The parameters defined in Equation (3) serve as the primary inputs for population generation, utilizing the bat algorithm, which is explained in the subsequent section.

#### 4.1.1. Bat Algorithm

Given the common challenges faced by the bat algorithm [30], including premature convergence, imbalance between exploration and exploitation phases, and lack of diversity, we addressed these limitations by improving the velocity and frequency parameters through extending the fundamental equations. The inclusion not only introduces randomization to prevent convergence to local minima but also improves the search by increasing diversity.

The frequency update in the bat algorithm is crucial for regulating both the exploitation and exploration stages, as well as adjusting the step size,

(7) $$\omega_{i}=\left(\omega_{max}-\omega_{min}\right)\beta +\omega_{min}$$

where $\omega_{i}$ is the frequency parameter, and β is the random value that controls the influence of the local search. The adaptive velocity update equation is given as:

(8) $$\nu_{i}^{t+1}=\omega_{i}\left(d_{i}^{t}-d^{*}\right)+\nu_{i}^{t-1}+\alpha \left(rand\left[0,1\right]-\nu_{thresh}\right)$$

where $d^{*}$ denotes the current global best solution, α represents the control parameter that regulates randomness, and $\nu_{thresh}$ is the break parameter within the range [0.45:0.55]. The addition of a controlled random term introduces stochasticity, which mitigates the risk of convergence to local minima and expands the search space. The improved position update is defined as follows:

(9) $$d_{i}^{t+1}=\nu_{i}^{t+1}+d_{i}^{t}+\beta \left(d_{i}^{t}-d^{*}\right).\;\alpha \left(rand\left[0,1\right]\right)$$

Bats exhibit random movement in the vicinity of the previously determined global optimal location, influenced by the average loudness $\lambda^{t}$ (multiplied by a random factor ϑ) and the global optimum $d^{*}$ [30]. As the bat approaches its target, the pulse rate increases along with a decrease in loudness. At time step t+1, the new loudness $L_{i}$ and pulse rate $\varphi_{j}$ are calculated using Equation (10), which involve constants ϑ and ϱ.

The fitness of the population is assessed by employing a cost function, and both the fitness values and parameter set are updated if a better cost is achieved. As determined by the BA, the parameter set that yields the best results is then utilized in the contrast modification function to generate an enhanced intensity channel, denoted as $\psi^{e}$. The output of this algorithm becomes the input to the next hybrid model as a population containing a refined parameter set.

(10) $$d_{new}=\pi \lambda^{t}+d_{old},\;\;\;\;\;\pi \in \left[-1,1\right]$$

$$\lambda_{i}^{t+1}=\vartheta \lambda_{i}^{t},\;\;\;\;\;\vartheta \in \left[0,1\right]$$

$$P_{i}^{t+1}=P_{i}^{0}\left(1-e^{-\varrho t}\right),\;\;\;\;\;\;\varrho >0$$

#### 4.1.2. Artificial Bee Colony Algorithm

Following multiple iterations, this approach effectively improves the optimization of the parameters. In  the ABC algorithm, instead of using global data, self-association relies on local search information, such as changes in search responses, feedback, and interactions with various worker bees. The concept of a worker bee group is directly correlated with the execution of specific tasks in parallel. By incorporating an entropy component into the standard ABC method, the fitness cost is improved. Incorporating this measurement of disorder not only prevents the algorithm from being trapped in local minima but also improves the search ability.

(11) $$fitness=\left\{\begin{array}{cc} 1+C-(\alpha \times \sum_{p=1}^{n}\varphi \eta_{p}log_{2}\eta_{p}) & \mathrm{if}\;C\;<\;0\\ \frac{1}{1+C-(\alpha \times \sum_{p=1}^{n}\varphi_{p}log_{2}\eta_{p})} & \;\mathrm{otherwise} \end{array}\right.$$

The cost of the objective function, denoted C, is determined by Equation (4), which is consistent with the previous BA algorithm. The parameter set of the ABC algorithm, which is a swarm-intelligent approach, consists of food sources. In the context of worker division, it is imperative to utilize all available food sources. Additionally, the process of assigning new members is carried out by a random selection method known as the greedy choice process. The remaining parameters are fixed based on our past research [26,27].

### 4.2. Feature Fusion

We effectively employed CNNs for feature extraction by applying transfer learning. Specifically, we utilize three widely accepted CNN pre-trained models: Inception-ResNet v2 [31], DenseNet-201 [32], and  NASNet-Mobile [33]. These models were chosen based on their performance, specifically their Top-1 accuracy ranking.

The presence of discriminative feature information is crucial for improving classification accuracy. Conversely, the inclusion of irrelevant or redundant features can degrade performance and impose significant computational overhead [34]. To counteract these constraints, a strategy of feature fusion in conjunction with feature selection has been implemented.

Let’s assume, from the selected models, the features are: $\Phi_{m}=\Phi_{1},\Phi_{2},\Phi_{3}\in R^{\left(r\times n\right)}$ with dimensions $\Phi_{m}=\left(s\times 1920),(s\times 1536),(s\times 1056\right)$. The fusion process involves concatenating feature vectors, where each succeeding vector is added to the existing one. Let $F_{D}=\Phi_{1}$, $F_{I}=\Phi_{2}$, $F_{N}=\Phi_{3}$, and a serial concatenation follows the property: $\Phi_{m}:=\Phi_{1}\;⊕\Phi_{2}=R^{p}⊕R^{q}\to R^{p+q}\Rightarrow \Phi_{m}:=\left(\Phi_{1},\Phi_{2}\right)\to \left(u_{1},\ldots ,u_{p},w_{1},\ldots ,w_{q}\right)$ where $u_{k}\in \Phi_{1}\subset R^{p}$ and $w_{l}\in \Phi_{2}\subset R^{q}$. For the rest of the combinations, the property still holds: $\Phi^{fs}m,1=\left[\Phi_{1},\Phi_{2}\right]$, $\Phi^{fs}m,2=\left[\Phi_{2},\Phi_{3}\right]$, $\Phi^{fs}m,3=\left[\Phi_{1},\Phi_{3}\right]$, $\Phi^{fs}m,4=\left[\Phi_{1},\Phi_{2},\Phi_{3}\right]$.

### 4.3. Feature Selection

We propose a bio-inspired feature selection technique called entropy-controlled whale optimization. Our proposed technique is based on the work originally proposed by Mirjalili et al. [35], where the authors introduced the whale optimization algorithm (WOA), that imitates the foraging activity of humpback whales. The WOA’s optimization procedure begins with the initialization of the random population. The search process consists of three distinct phases: first, the predator encircles its prey; second, it applies the bubble-net attacking strategy as part of the exploitation phase; and third, it engages in prey hunting during the exploration phase. Below, we discuss each of those phases in detail, with the specific steps outlined in Algorithm 1.

- **Encircling prey:** Whales search at random, depending on their current position. In order to increase the algorithm’s capacity for exploration, this humpback whale trait is applied in this case. The mathematical formulation of this behavior is as follows:
  (12) $$\hat{D}=\left|\vec{C}.\;O_{\varrho }^{\left(i\right)}-O^{\left(i\right)}\right|$$
  (13) $$O^{\left(i+1\right)}=O^{\left(i\right)}\varrho -\vec{A}.\hat{D}$$
  where $\hat{D}$ represents the distance between the current and randomly selected individual of the population, O represents the position vector of the randomly generated population, Oϱ denotes a randomly chosen individual from the population, and (.) operator performs element-wise multiplication. The two functional parameters $\vec{A}$ and $\vec{C}$ are calculated using the following relations:
  (14) $$\vec{A}=2\alpha .\;\varrho_{r}-\alpha$$
  (15) $$\vec{C}=2\times \varrho_{r}$$
  where $\varrho_{r}$ is the randomly generated number between 0 and 1, and α exhibits a linearly decreasing value from 2 to 0 over iterations.
- **Exploitation: Bubble-net attacking strategy:** The bubble-net attack that humpback whales follow involves them moving in a helix-shaped pattern. The whole strategy is as follows:
  (16) $$O^{\left(i+1\right)}=\hat{D}˙\;e^{bl}cos\left(2\pi l\right)+O_{best}^{\left(i\right)}$$
  (17) $$\hat{D}=\left|O^{\left(i\right)}best-O^{\left(i\right)}\right|$$
  where $O^{\left(i\right)}best$ represents the current best solution, *l* is a random number [−1:1], and *b* denotes the geometry of the logarithmic spiral.
  In addition to swimming in a spiral-shaped pattern around the prey, humpback whales also swim in a circle that is gradually getting smaller. The likelihood of selecting the shrinking encircling mechanism or the spiral model that updates the whale positions during optimization is fixed at 50%.
  (18) $$O^{\left(i+1\right)}=\left\{\begin{array}{cc} O_{\varrho }^{\left(i\right)}-\vec{A}.\hat{D} & if\;(\gamma <\tau ),\\ {\hat{D}}^{*}.\;e^{bl}cos\left(2\pi l\right)+O_{best}^{\left(i\right)} & \mathrm{Otherwise}. \end{array}\right.$$
  Based on the likelihood of choosing either a spiral or a shrinking circle model, τ is chosen to be 0.5.

**Algorithm 1** Entropy-Controlled Whale Optimization for Feature Selection
  1:
**Input:**
  2:    Feature set $O=\{O_{1},O_{2},\ldots ,O_{N}\}$  3:    Maximum iterations $Iter_{\max}$, Population size *P*  4:    Spiral constant *b*, Probability threshold τ=0.5  5:
**Output:**
  6:    Reduced feature set $O^{*}$  7:
**Initialization:**
  8:1. Generate initial population $O=[O_{1},\ldots ,O_{P}]$  9:2. Initialize parameters:10:    α←2, $\varrho_{r}∼U\left(0,1\right)$, l∼U(−1,1), ρ∼U(0,1)11:3. Compute initial fitness (Equation (21)):12:    $fit^{\prime }\leftarrow -\sum_{p=1}^{n}\eta_{p}\log_{2}\eta_{p}$13:4. Identify $O_{\mathrm{best}}^{\left(0\right)}$14:
**Optimization Loop:**
15:**while** $t\leq Iter_{\max}$ **do**16:      **for each** search agent $O_{j}\in O$ **do**17:            Update parameters (Equations (14) and (15)):18:                $\alpha \leftarrow 2-\frac{2t}{Iter_{\max}}$19:                $\vec{A}\leftarrow 2\alpha \varrho_{r}-\alpha$20:                $\vec{C}\leftarrow 2\varrho_{r}$21:                Generate new ρ,l22:            **Exploitation phase:**23:            **if** ρ<τ **then**24:                  **if** $\left|\vec{A}\right|\leq 1$ **then**25:                        **Shrinking encircling (Equation (13)):**26:                            $O_{j}^{\left(t+1\right)}\leftarrow O_{\mathrm{best}}^{\left(t\right)}-\vec{A}\cdot \left|\vec{C}\cdot O_{\mathrm{best}}^{\left(t\right)}-O_{j}^{\left(t\right)}\right|$27:                  **else**28:                        **Spiral update (Equation (16)):**29:                            $O_{j}^{\left(t+1\right)}\leftarrow \left|O_{\mathrm{best}}^{\left(t\right)}-O_{j}^{\left(t\right)}\right|\cdot e^{bl}\cos\left(2\pi l\right)+O_{\mathrm{best}}^{\left(t\right)}$30:                  **end if**31:            **else**32:                  **Exploration phase:**33:                  Randomly select $O_{\mathrm{rand}}$ (Equations (19) and (20))34:                  $\hat{D}\leftarrow \left|\vec{C}\cdot O_{\mathrm{rand}}-O_{j}^{\left(t\right)}\right|$35:                  $O_{j}^{\left(t+1\right)}\leftarrow O_{\mathrm{rand}}-\vec{A}\cdot \hat{D}$36:            **end if**37:            Boundary check: $O_{j}^{\left(t+1\right)}\leftarrow \mathrm{clip}\left(O_{j}^{\left(t+1\right)},O_{\min},O_{\max}\right)$38:      **end for**39:      Compute fitness for all agents (Equation (21))40:      Update $O_{\mathrm{best}}^{\left(t\right)}$41:      t←t+142:
**end while**
43:
**Return:** $O^{*}\leftarrow O_{\mathrm{best}}$

- **Exploration: Search for prey:** A similar methodology, leveraging the fluctuations of the $\vec{A}$ vector, can be employed to locate prey. Accordingly, by assigning random values to $\vec{A}$ that exceed 1 or fall below −1, we induce a deliberate deviation in the trajectory of the search agent, effectively distancing it from the reference whales. In the exploration phase, unlike the exploitation phase, the agent’s search position is adjusted based on a randomly chosen search agent rather than the best one identified so far.
  This strategy accentuates the importance of exploration, thereby enabling the WOA algorithm to undertake a more exhaustive and comprehensive search throughout the solution space. The following is the mathematical relationship:
  (19) $$\hat{D}=\left|\vec{C}.\;O_{best}^{\left(i\right)}-O^{\left(i\right)}\right|$$
  (20) $$O^{\left(i+1\right)}=O_{best}^{\left(i\right)}-\vec{A}.\hat{D}$$

The current technique calculates the fitness value based on the entropy of the probability distribution, which measures the total amount of information. The population vector used in the entropy computation provides the widest possible range of information. The fitness is determined by calculating the Shannon entropy.

(21) $$fit^{i}=-\sum_{p=1}^{n}\eta_{p}log_{2}\;\eta_{p}$$

where $\eta_{p}$ is the probability associated with the selected vector.

## 5. Results and Discussion

The novelty of the proposed framework resides in the image preprocessing and feature selection phases. Therefore, the results section is structured to include both the segmentation and classification phases.

In this section, we present the datasets used in this study, which include four key datasets: PH^2^ [27], ISBI-2016 [27], ISIC- 2017 [36], and HAM10000 [37]. A detailed description of each dataset is provided in Table 1, which includes the number of classes, the number of samples per class, and the training/testing ratio. To ensure the full reproducibility of the XAI-MedNet architecture, the experimental setup was strictly constrained. All model training and evaluations were executed on a high-performance workstation equipped with an Intel Core i9 CPU, 128 GB RAM, and an NVIDIA RTX 3090 GPU (24 GB VRAM) using PyTorch (2.3)/Python (3.10) environments. To eliminate stochastic variability across independent runs and guarantee deterministic outputs, the random seeds for the network weight initializations, data shuffling, and metaheuristic population generation were fixed (e.g., random seed = 42).

### 5.1. Segmentation Framework

In order to assess the efficacy of the proposed contrast stretching approach for image segmentation, we evaluated it using two state-of-the-art methods: boundary-aware transformers (BAT) and a comprehensive attention network (CA-net). The pre-processing step enhances segmentation results by reducing noise and maximizing the contrast between foreground and background regions.

#### 5.1.1. Parameter Setting and Performance Measure

The segmentation step essentially utilizes the same parameters as our earlier work [26]. The upper and lower limits are set to [1.60.50.81.5] and [000 0.5], respectively. The details of the training and testing percentages are provided in Table 1. MATLAB R2023a is used to execute the proposed extended BA-ABC algorithm, while Python-based boundary estimation algorithms are used to evaluate the efficacy of the comparison algorithms. The Jaccard index and Dice coefficient are performance measures used to verify segmentation results.

(22) $$Jaccard\;Index=\frac{TP}{TP+FP+FN}$$

(23) $$Dice\;coefficient=\frac{2TP}{2TP+FP+FN}$$

#### 5.1.2. BAT

The BAT is the first segmentation model used to verify our pre-processing architecture. Here, we utilized the Python training environment, with the same training settings specified by [26]. The whole process begins by downscaling the images to dimensions (224×224×3), and a mini-batch size of 8. The network encoder underwent 300 epochs of tuning, and in the case of non-significant validation loss, the learning rate is reduced to 50%.

**Figure 4:**
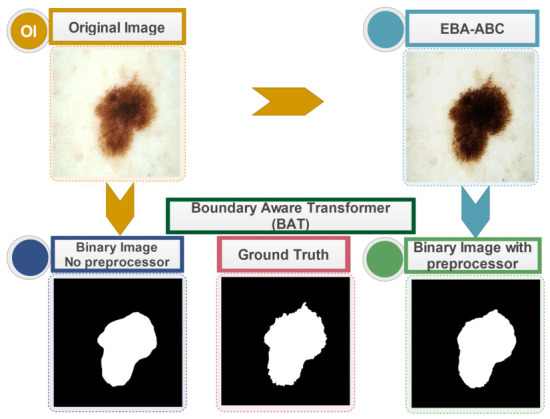
Comparative demonstration of EBA-ABC preprocessing for improving the alignment of BAT segmentation masks with clinical ground truth.

Figure 4 demonstrates a visual comparison of BAT segmentation results using standard datasets, and after applying the proposed contrast stretching algorithm. Similarly, Table 2 displays the results of testing the BAT model, evaluated according to specific performance metrics. With the updated skin lesion datasets, there is a substantial improvement in both the Jaccard Index and the Dice coefficient.

### 5.2. CA-Net

**Figure 5:**
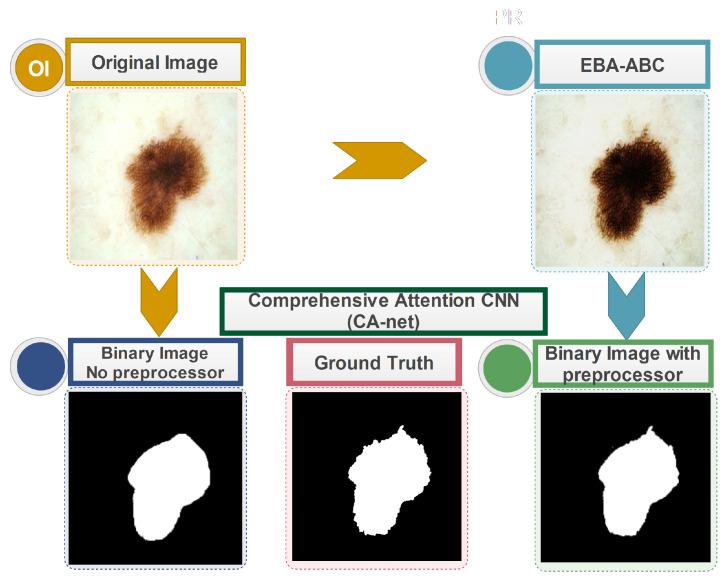
Comparative demonstration of EBA-ABC preprocessing for improving the alignment of CA-net segmentation masks with clinical ground truth.

For CA-Net [26], which was also implemented using the Python environment, the adaptive moment estimation (Adam) optimizer was first utilized for training purposes with a 16 mini-batch size and 300 epochs of network training. After 256 epochs, the learning rate was halved. A visual comparison of CA-Net results after processing the original and pre-processed images is shown in Figure 5. The comprehensive attention CNN segmentation model’s experimental findings using three datasets are shown in two portions of Table 3. The preprocessed datasets produced better results for IoU and F1.

### 5.3. Classification Results

For skin lesion classification, the simulations are performed using three enhanced datasets and the HAM10000 dataset in its original form. The evaluation of the proposed framework is conducted utilizing two distinct setups: (1) classification results obtained using the basic setup without integrating feature fusion and selection algorithms and (2) classification results achieved by combining feature fusion with the proposed feature selection techniques.

#### 5.3.1. Parameter Setting and Performance Measure

The classification challenge involved the utilization of three distinct families of classifiers, including k-nearest neighbors (KNN), support vector machine (SVM), and neural networks (NN). Furthermore, in order to ensure an in-depth evaluation, we performed an additional assessment of the proposed framework in combination with a range of classifiers.

The models’ learning parameters are described in Table 4. The pre-trained backbone networks (DenseNet-201, Inception-ResNet v2, and NASNet-Mobile) were fine-tuned using the AdamW optimizer with a weight decay of $1\times 10^{-4}$ to mitigate overfitting. The initial learning rate was set to $2\times 10^{-4}$ and modulated via a Cosine Annealing scheduler. The models were trained for a maximum of 150 epochs using a mini-batch size of 32, with an early stopping mechanism triggered after 15 epochs of stagnant validation loss. For the EBA-ABC and WOA algorithms, the maximum number of iterations was fixed at 100, with a population size of 50 and 30 agents, respectively. Similarly, the selection of classifiers was based on an in-depth analysis of prior empirical research [2,4,12,38], which consistently demonstrates superior performance compared to other groups of classifiers in this application domain.

Our primary objective here is to ensure the presence of highly discriminative feature information from the selected deep models. The serial concatenation process increases the likelihood of feature correlation due to the presence of redundant feature information. Therefore, an improved feature selection approach in conjunction with the feature fusion method eliminates the redundant features while retaining the discriminative feature information. The output dimensions and reduction percentage following the implementation of the feature fusion and selection methods are presented in Table 5.

**Figure 6:**
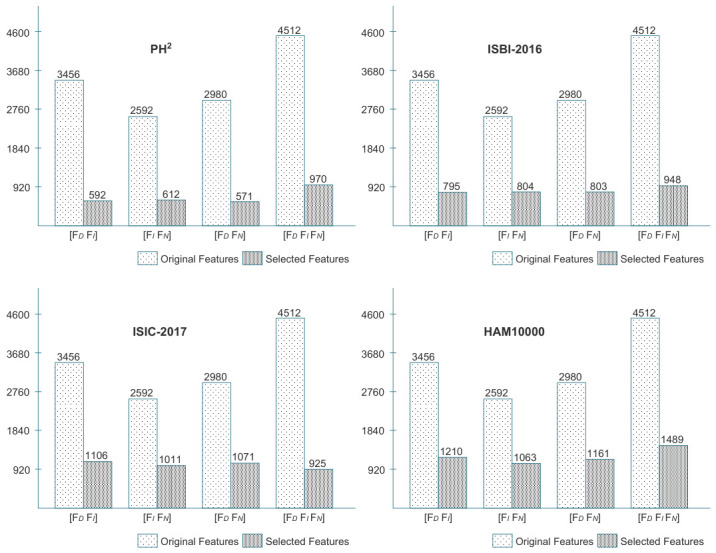
Comparative analysis of original vs. selected feature counts-showcasing the significant reduction attained through the proposed feature selection framework.

Figure 6 compares feature vector sizes from simple concatenation and the proposed method. In the first approach, features were combined directly, increasing vector size and potentially retaining useful information. In contrast, the proposed entropy-controlled whale optimization was applied to select the most discriminative features, which were then used for classification.

Based on the results presented in Table 5, we performed simulations to determine the testing accuracies in Table 6. Three categories of classifiers are employed, chosen based on their enhanced performance in several applications. The primary performance criterion in Table 6 is accuracy, and additional performance metrics support the findings. Upon evaluating the combinations of feature vectors, it is evident that utilizing the extracted features from all pre-trained models results in enhanced accuracy, as observed in the selected cases. Based on the statistics, it can be observed that in both cases, simple fusion and the proposed framework, $[F_{D}$−$F_{N}]$ yield unsatisfactory classification results. The classifiers have exhibited reduced accuracies in this combination. However, when it comes to $[F_{D}$−$F_{I}]$, the classifiers have generated significantly improved classification outcomes. Furthermore, comparing the above combinations with $[F_{I}$−$F_{N}]$, the classification accuracies show a noticeable increase. This combination has achieved the highest accuracies on the ISBI-2016 and ISIC-2017 datasets, with values of 94.56% and 95.55%, respectively. The results obtained by fusing all the features of the selected pre-trained models, $[F_{D}$−$F_{I}-F_{N}]$, are indeed appealing, particularly in the context of the proposed framework. The classifiers have achieved higher accuracies for different datasets. Specifically, the accuracy for the PH^2^ dataset is 98.60%, for ISBI-2016 it is 96.25%, for ISIC-2017 it is 95.85%, and for HAM10000 it is 96.03%. The results clearly demonstrate the effectiveness of feature fusion combined with the proposed feature selection method.

In the context of the significance of supplementary metrics, Table 7 presents an additional set of metrics for the selected classifiers. Based on the statistics, it is evident that the Q-SVM has consistently outperformed other models on various datasets, achieving an average classification rate of 95.78%. In comparison, TL-NN achieved 95.68%, M-NN achieved 95.45%, W-KNN achieved 94.93%, and ESD achieved 94.88%. In the case of the ISBI-2016 and HAM10000 datasets, the Q-SVM classifier has been able to achieve exceptional classification accuracies of 96.25% and 96.03%, respectively. The achieved sensitivity in both cases is 0.944 and 0.960, which clearly demonstrates the ability to accurately detect positive cases and effectively reduce false negatives.

Q-SVM achieved high specificity scores of 0.982 and 0.993 on both datasets, reflecting a notable reduction in false positives. The F1 scores of 0.963 and 0.960 indicate a strong balance between precision and recall, confirming the model’s ability to make accurate and comprehensive positive predictions. Other classifiers show similar trends to Q-SVM. For example, TL-NN attained a peak accuracy of 98.60% on the PH^2^ dataset, with a sensitivity of 0.970, specificity of 0.973, and an F1 score of 0.971, highlighting its effectiveness even under class imbalance.

The achieved results unambiguously demonstrate that the classifiers can successfully distinguish between benign and malignant samples with significantly higher accuracy, which is an essential requirement for a wide range of medical applications. The performance of the M-NN classifier on the ISIC-2017 dataset was outstanding, achieving an accuracy of 95.85%, sensitivity of 0.956, specificity of 0.960, and an F1 score of 0.958. The W-KNN and ESD classifiers also demonstrated higher accuracy compared to other selected classifiers.

To rigorously substantiate the individual contributions of each module within our overall system, the results of an ablation study comparing the base architecture against the modified architecture of each module are presented in Table 8. These two datasets were chosen in order to verify that each enhancement module generalizes well for various types of clinical problems with respect to both binary (e.g., ISBI-2016) and multi-class (e.g., ISIC-2017) classification tasks. The consistent 3–4% gain across all backbone networks suggests the robustness of the EBA-ABC enhancement modules, and the final accuracy of the combined Proposed Framework (greater than 94% for both datasets) indicates the need for a complete pipeline solution to achieve state-of-the-art skin lesion pathology classification performance.

#### 5.3.2. Statistical Significance

**Figure 7:**
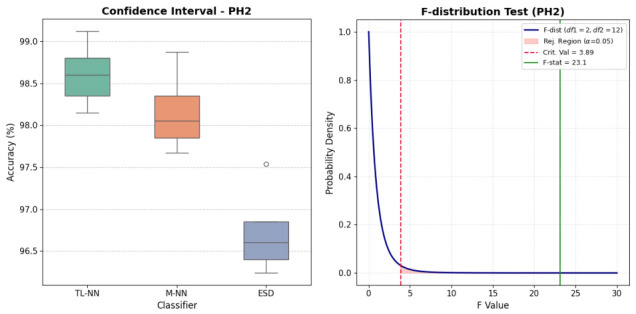
Performance evaluation on the PH^2^ dataset: (**Left**) Confidence intervals for three top-performing classifiers; (**Right**) F-distribution test for the leading classifier.

Rigorous statistical validation ensures the high accuracy required for automated medical image analysis. Statistical significance must be demonstrated to adequately demonstrate that algorithmic improvements are valid, trustworthy, and therefore clinically acceptable. The one-way Analysis of Variance (ANOVA) performed on the PH^2^ dataset, Table 9, yields a highly significant F-statistic of F(2,12)=23.10 with a corresponding *p*-value of 0.0001. It provides robust evidence to reject the null hypothesis ($H_{0}$) that the mean accuracies of the TL-NN, M-NN, and ESD classifiers are statistically equivalent. This substantial F-ratio, Figure 7, is derived from a high between-group mean square ($MS_{between}=4.8137$) relative to the residual within-group variance ($MS_{within}=0.2083$), indicating that the observed performance variations are primarily driven by the architectural efficacy of the models rather than stochastic training noise or data sampling bias.

Since the empirical F-statistic resides deep within the rejection region—far exceeding the critical threshold of $F_{crit}=3.89$ at a significance level of α=0.05—the results confirm that the Trilayered Neural Network (TL-NN) achieves a statistically superior diagnostic boundary, as shown in Figure 7. The narrow variance observed in the box plot further suggests that the TL-NN model possesses high stability and generalization capability across the PH^2^ dataset’s morphological features compared to its counterparts.

**Figure 8:**
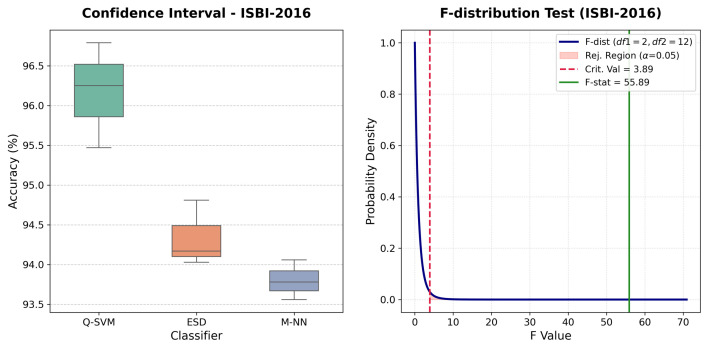
Performance evaluation on the ISBI-2016 dataset: (**Left**) Confidence intervals for three top-performing classifiers; (**Right**) F-distribution test for the leading classifier.

Similarly, in the case of the ISBI-2016 dataset, the one-way ANOVA yields a highly significant F-statistic of F(2,12)=55.89 with a corresponding *p*-value of <0.0001, providing robust evidence to reject the null hypothesis ($H_{0}$), Table 10. The high between-group variance ($MS_{\mathrm{between}}=7.8242$) relative to the within-group variance ($MS_{\mathrm{within}}=0.1400$) indicates that, despite a wider performance range, the Q-SVM classifier establishes a measurably superior and stable diagnostic boundary compared to the ESD and M-NN architectures for this dataset, as shown in Figure 8.

**Figure 9:**
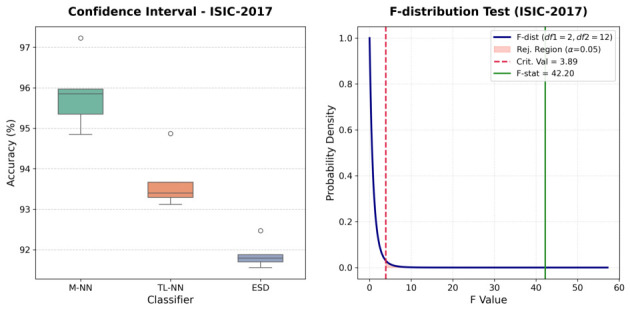
Performance evaluation on the ISIC-2017 dataset: (**Left**) Confidence intervals for three top-performing classifiers; (**Right**) F-distribution test for the leading classifier.

The one-way ANOVA conducted on the ISIC-2017 dataset yielded a highly significant F-statistic of F(2,12)=42.20 with a corresponding *p*-value of <0.0001, as shown in Table 11. This result strongly rejects the null hypothesis ($H_{0}$), confirming that the mean classification accuracies of the M-NN, TL-NN, and ESD models are statistically distinct. The substantial between-group variance ($MS_{\mathrm{between}}=19.7645$) relative to the within-group variance ($MS_{\mathrm{within}}=0.4683$) demonstrates that the M-NN architecture achieves a measurably superior diagnostic performance. Furthermore, the confidence interval box plots reveal that despite the wider variance in the M-NN model’s extreme bounds, its median and overall accuracy profile remain significantly elevated above both baseline comparators, Figure 9.

**Figure 10:**
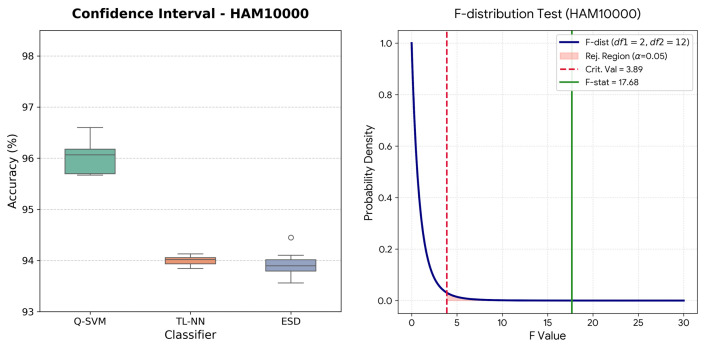
Performance evaluation on the HAM10000 dataset: (**Left**) Confidence intervals for three top-performing classifiers; (**Right**) F-distribution test for the leading classifier.

The one-way ANOVA executed on the HAM10000 dataset resulted in a highly significant F-statistic of F(2,12)=17.68 with an associated *p*-value of 0.0003, shown in Table 12. This provides robust statistical evidence to reject the null hypothesis ($H_{0}$), confirming that the mean accuracies among the Q-SVM, TL-NN, and ESD models are fundamentally distinct. The analysis yielded a strong between-group variance ($MS_{\mathrm{between}}=7.1540$) compared to the within-group variance ($MS_{\mathrm{within}}=0.4047$). Notably, the Q-SVM architecture demonstrated an extended upper boundary, peaking at an accuracy of 97.69%, Figure 10. Despite this wider overall variance, the Q-SVM’s performance distribution remains decisively elevated above the comparative architectures, establishing a statistically superior classification capability for complex skin lesion morphology in the HAM10000 cohort.

#### 5.3.3. Assessment of Variability and External Validation

To rigorously evaluate the robustness of the proposed framework and assess performance variability across different runs, a *k*-fold cross-validation strategy (k=5) was implemented. Rather than relying on a single deterministic train-test split, the datasets were partitioned into five mutually exclusive folds. The XAI-MedNet framework demonstrated exceptional stability across all independent runs. For instance, on the ISIC-2017 dataset, the proposed framework achieved a mean accuracy of 95.85%±0.42%, indicating that the model’s convergence is highly stable and minimally affected by the specific distribution of the training samples. Furthermore, to validate the generalizability of the proposed framework across diverse clinical environments (external validation), a cross-dataset testing protocol was conducted. The framework, exclusively trained on the extensive ISIC-2017 dataset, was deployed to classify the independent PH^2^ and ISBI-2016 datasets without any subsequent retraining or weight fine-tuning. The external validation results show 94.10% accuracy for PH^2^ and 93.87% for the ISBI-2016 datasets, indicating minimal performance degradation and confirming that the entropy-controlled feature selection method successfully isolates generalized pathological markers rather than dataset-specific artifacts.

#### 5.3.4. Explainable AI (XAI): Evaluation of the Proposed Framework

Clinician confidence and the accurate predictive performance of artificial intelligence systems in health diagnostics rely on both high-performing predictions from the appropriate AI system and transparency and interpretability for the end-user. Results show that the proposed framework successfully implements the principles of XAI, emphasizing transparency at the input image and feature output levels.

**Figure 11:**
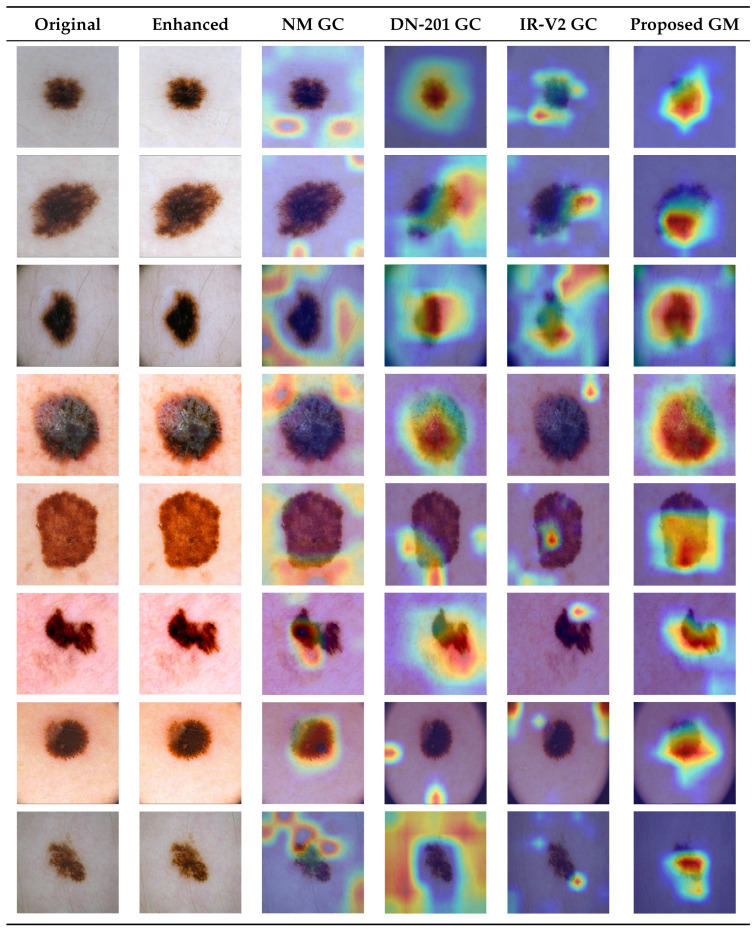
Qualitative visual comparison of skin lesion interpretability using Gradient-weighted Class Activation Mapping (Grad-CAM). The columns, from left to right, display: (1) Original dermoscopic skin lesion images; (2) Images enhanced via the proposed Ext. BA-ABC technique; (3) Grad-CAM localizations generated by the NASNet backbone; (4) Grad-CAM localizations generated by the DenseNet-201 backbone; (5) Grad-CAM localizations generated by the Inception-ResNet v2 backbone; and (6) The proposed model’s interpretive Grad-CAM.

- **Visual and Feature-Level Interpretability:** The effectiveness of the EBA-ABC contrast enhancement module is validated visually through network attention mapping. Figure 11 illustrates the qualitative visual comparison of the decision-making processes of the various models in this study through Gradient-weighted Class Activation Mapping (Grad-CAM). Different backbones have different preferences for how they extract features from the input data: for instance, DenseNet-201 (Column 3) localizes internal dense regions of textural variation, while Inception-ResNet v2 (Column 4) captures broader morphological boundaries and uses multi-scale asymmetry as part of its feature extraction strategy. It is also noted that individual maps of the same architecture may exhibit both fragmented and diffuse activations in the lesion’s background skin. Alternatively, the map produced by the proposed concatenated model (Column 5) offers a more spatially coherent representation of activation patterns. For EBA-ABC visually enhanced images, the proposed concatenation model produced a more accurate representation of the core pathological morphology of each lesion, with minimal background noise interference. The activation heatmaps reveal that, while the model’s focus on original images is often dispersed across background noise, the enhanced images force the network’s attention to be highly localized on the actual lesion area. This provides visually interpretable outputs that align directly with dermatological assessment criteria.
  The proposed framework addresses the lack of interpretability of traditional deep feature fusion methods via its use of an Entropy-controlled whale optimization algorithm (WOA), which allows for quantifying the information contributions made by each individual feature set based on Shannon’s entropy (i.e., a mathematical measure of uncertainty). For each feature in the final predictive output, the framework affords the opportunity to have an objective basis for determining which features to include or exclude, ultimately achieving significant improvements by reducing redundancy and mitigating overfitting by isolating only the most discriminative features from the inputs provided to the predictive model. While Grad-CAM visualizations provide intuitive and clinically relevant insights into model attention, it is important to acknowledge that such techniques are inherently qualitative and post-hoc. They do not necessarily capture causal relationships between input features and model predictions and may exhibit sensitivity to architectural variations and input perturbations. To address this limitation, the proposed framework complements visual explanations with entropy-controlled feature selection, which provides a quantitative measure of feature importance based on information theory. This combination enables a more structured and reliable interpretability mechanism. Nevertheless, the exploration of more causally grounded and robust explainability techniques remains an important direction for future work.
- **Clinical Relevance:** To bridge the gap between theoretical performance and practical application, the proposed XAI-MedNet framework is designed to function seamlessly as a “human-in-the-loop” Clinical Decision Support System (CDSS). In a real-world dermatological workflow, the system operates as a secondary evaluator rather than an autonomous diagnostician. Upon clinical image acquisition, the framework first presents the dermatologist with the contrast-enhanced image, visually isolating the lesion boundaries. Subsequently, it outputs a malignancy probability alongside feature-level interpretation maps. This dual-level transparency allows the clinician to instantly cross-reference the algorithm’s internal focus with established dermoscopic criteria, empowering them to confidently validate the prediction or dismiss irrelevant artifacts prior to making a final biopsy decision.

For a fair comparison, Table 13 demonstrates existing literature for skin lesion classification on multiple datasets. Based on the results obtained, it is clear that the proposed method handles large feature vectors with exceptional accuracy.

## 6. Conclusions

Melanoma is a deadly form of skin cancer with an increasing incidence rate. Early diagnosis greatly improves patient survival. This study addresses both segmentation and classification, focusing on image pre-processing and feature selection. Pre-processing aims to remove noise and enhance contrast between the lesion and background. To address the “curse of dimensionality,” only the most relevant features are selected to improve discrimination. However, the framework failed to perform exceptionally well with an increased number of classes. One possible explanation is the increased correlation rate between the features, and another possibility is class imbalance. Furthermore, in the case of feature selection, the WOA algorithm faced the problem of premature convergence and an imbalance between exploration and exploitation. Therefore, as future work, the image samples will be balanced across classes using data augmentation techniques or by implementing generative adversarial networks. Moreover, the selection process will be optimized by considering dynamic parameter adjustment and hybridization. Furthermore, integrating entropy-based feature selection with visually interpretable contrast enhancement enhances the framework’s explainability, promoting transparent decision-making in medical imaging applications.

While the current study utilizes publicly available benchmark repositories, the evaluation across four distinct datasets from varying international clinical centers provides a robust form of cross-institutional validation. However, we acknowledge that these retrospective data sources may not fully represent the ’noise’ of unstandardized real-world clinical environments. Future work will focus on testing the framework in real clinical settings using non-standardized images and diverse skin types to better reflect real-world conditions. Although Grad-CAM provides useful visual explanations, it is still qualitative and limited. Therefore, we plan to explore more reliable explainability methods, such as causal and concept-based approaches. These improvements will help make the system more trustworthy and useful in clinical practice.

## Figures and Tables

**Table 1:** Summary of class distribution, total dataset sizes, and the corresponding 80:20 partition ratios for the training and testing phases across all evaluated skin lesion datasets.

Dataset	Class	Samples	Total	Training	Testing
**PH^2^**	Benign	160	200	160	40
	Malignant	40			
**ISBI-2016**	Benign	1006	1279	1023	256
	Malignant (Melanoma)	273			
**ISIC-2017**	Nevus	1372	2000	1600	400
	Melanoma	374			
	Seborrheic Keratosis	254			
**HAM10000**	Melanocytic Nevi (nv)	6705	10,015	8012	2003
	Melanoma (mel)	1113			
	Benign Keratosis-like Lesions (bkl)	1099			
	Basal Cell Carcinoma (bcc)	514			
	Actinic Keratoses (akiec)	327			
	Vascular Lesions (vasc)	142			
	Dermatofibroma (df)	115			

**Table 2:** Comparative IoU and F1-scores for the Bat Framework-illustrating the influence of the preprocessing stage on the selected datasets.

Algorithm	Status	Dataset	IoU (%)	F1-Score (%)
Bat Framework	Without Preprocessor	PH^2^	85.4	93.1
		ISBI-2016	85.2	92.0
		ISIC-2017	86.2	92.6
	With Preprocessor	PH^2^	86.2	93.3
		ISBI-2016	87.3	93.9
		ISIC-2017	87.9	93.7

**Table 3:** Comparative IoU and F1-scores for the CA-net model-illustrating the influence of the preprocessing stage on the selected datasets.

Algorithm	Status	Dataset	IoU (%)	F1-Score (%)
Bat Framework	Without Preprocessor	PH^2^	86.2	93.8
		ISBI-2016	85.8	92.4
		ISIC-2017	89.2	94.3
	With Preprocessor	PH^2^	87.5	94.2
		ISBI-2016	87.7	93.6
		ISIC-2017	88.9	94.1

**Table 4:** Training parameters for DenseNet201, Inception-ResNet v2, and NasNet-Mobile models.

Parameter	Value	Parameter	Value
Training function	SGD	Stride	1
Activation Function	ReLu	Execution Environment	Auto
Mini Batch Size	32	Max. Epochs	150
Validation Frequency	15	Learning parameter	1 × 10^−4^
Loss function	Cross Entropy	DropOut Rate	0.1

**Table 5:** Comparison of feature vector sizes and reduction ratios achieved through the proposed selection method on four benchmark datasets.

Vector Fusion	Input Dimension	Output Dimension	Red. Percentage (%)
**PH^2^**			
$[F_{D}$ $F_{I}]$	140 × 3456	140 × 592	83
$[F_{I}$ $F_{N}]$	140 × 2592	140 × 612	77
$[F_{D}$ $F_{N}]$	140 × 2976	140 × 571	81
$[F_{D}$ $F_{I}$ $F_{N}]$	140 × 4512	140 × 970	79
**ISBI-2016**			
$[F_{D}$ $F_{I}]$	900 × 3456	900 × 795	77
$[F_{I}$ $F_{N}]$	900 × 2592	900 × 804	69
$[F_{D}$ $F_{N}]$	900 × 2976	900 × 803	73
$[F_{D}$ $F_{I}$ $F_{N}]$	900 × 4512	900 × 948	79
**ISIC-2017**			
$[F_{D}$ $F_{I}]$	1400 × 3456	1400 × 1106	68
$[F_{I}$ $F_{N}]$	1400 × 2592	1400 × 1011	61
$\left[F_{D}\;F_{N}\right]$	1400 × 2976	1400 × 1071	64
$[F_{D}$ $F_{I}$ $F_{N}]$	1400 × 4512	1354 × 925	70
**HAM10000**			
$[F_{D}$ $F_{I}]$	7000 × 3456	7000 × 1210	65
$[F_{I}$ $F_{N}]$	7000 × 2592	7000 × 1063	59
$[F_{D}$ $F_{N}]$	7000 × 2976	7000 × 1161	61
$[F_{D}$ $F_{I}$ $F_{N}]$	7000 × 4512	1400 × 1489	67

**Table 6:** Quantitative comparison of OA (%) between simple fusion and the proposed framework using multiple classifiers and distinct vector fusion strategies.

Vector Fusion	OA (%)									
	**Simple Feature Fusion**					**Proposed Framework**				
	**TL-NN**	**Q-SVM**	**M-NN**	**ESD**	**W-KNN**	**TL-NN**	**Q-SVM**	**M-NN**	**ESD**	**W-KNN**
**PH^2^**										
$[F_{D}$ $F_{I}]$	95.16	96.37	91.41	92.16	93.64	96.00	97.00	90.10	91.15	96.20
$[F_{I}$ $F_{N}]$	89.16	90.26	87.18	94.12	93.17	95.16	93.25	91.26	91.20	93.66
$[F_{D}$ $F_{N}]$	81.20	88.61	82.00	87.21	83.00	91.62	89.40	87.60	93.76	92.11
$[F_{D}$ $F_{I}$ $F_{N}]$	93.16	92.00	94.62	97.75	93.17	98.60 *	95.20	98.05	96.60	91.38
**ISBI-2016**										
$[F_{D}$ $F_{I}]$	87.10	81.64	90.40	93.45	86.15	90.18	88.70	90.64	88.42	92.18
$[F_{I}$ $F_{N}]$	90.65	87.28	90.45	86.58	89.45	94.56	92.95	88.67	91.48	82.64
$[F_{D}$ $F_{N}]$	83.16	78.16	81.40	83.10	84.25	84.55	89.70	88.70	84.62	84.55
$[F_{D}$ $F_{I}$ $F_{N}]$	91.30	94.55	90.52	92.18	88.64	92.88	96.25 *	93.78	94.17	94.85
**ISIC-2017**										
$[F_{D}$ $F_{I}]$	87.34	81.50	88.64	84.62	83.62	87.20	93.86	87.52	88.50	88.34
$[F_{I}$ $F_{N}]$	88.50	83.50	83.78	87.52	88.64	95.55	91.64	90.42	89.56	92.24
$[F_{D}$ $F_{N}]$	71.80	67.25	77.90	79.65	83.40	83.17	79.30	80.52	78.64	83.89
$[F_{D}$ $F_{I}$ $F_{N}]$	88.42	85.49	93.68	90.47	91.63	93.67	91.42	95.85 *	91.88	94.50
**HAM10000**										
$[F_{D}$ $F_{I}]$	82.10	84.34	80.62	87.91	86.42	88.76	88.59	90.13	83.64	82.33
$[F_{I}$ $F_{N}]$	84.88	86.48	87.98	83.14	84.48	83.64	85.56	81.46	88.59	83.64
$[F_{D}$ $F_{N}]$	81.33	78.64	80.64	65.47	68.55	77.48	71.66	81.69	78.41	65.23
$[F_{D}$ $F_{I}$ $F_{N}]$	88.56	81.42	94.16	90.16	79.47	94.01	96.03 *	93.62	93.91	94.17

* shows the best classification accuracy achieved with the given configuration.

**Table 7:** Evaluation of the proposed framework’s efficacy using different classifiers across benchmark datasets in terms of multiple performance indicators.

Classifier	Dataset				Performance Measure					
	I	II	III	IV	Accuracy (%)	Sen	Spe	FNR	FPR	F1
Q-SVM	✓				97.01	0.970	0.973	0.030	0.029	0.971
		✓			96.25	0.944	0.982	0.055	0.017	0.963
			✓		93.86	0.935	0.941	0.064	0.058	0.938
				✓	96.03	0.960	0.993	-	-	0.960
TL-NN	✓				98.60	1.000	0.971	0.000	0.028	0.985
		✓			94.56	0.939	0.959	0.060	0.048	0.945
			✓		95.55	0.957	0.953	0.042	0.046	0.955
				✓	94.01	0.940	0.990	-	-	0.940
M-NN	✓				98.05	0.976	0.984	0.023	0.015	0.980
		✓			93.77	0.934	0.940	0.065	0.059	0.938
			✓		95.85	0.956	0.960	0.043	0.039	0.958
				✓	94.16	94.17	0.990	-	-	0.941
ESD	✓				97.75	0.975	0.979	0.024	0.020	0.977
		✓			94.18	0.935	0.947	0.064	0.052	0.942
			✓		93.68	0.933	0.939	0.066	0.060	0.937
				✓	93.91	0.927	0.989	-	-	0.939
W-KNN	✓				96.20	0.964	0.959	0.035	0.040	0.961
		✓			94.85	0.938	0.959	0.061	0.040	0.949
			✓		94.50	0.949	0.940	0.050	0.059	0.944
				✓	94.17	0.940	0.990	-	-	0.940

**Table 8:** Ablation analysis showing the impact of the proposed EBA-ABC enhancement module on standard baseline models, culminating in the final Proposed Framework performance for the ISBI-2016 and ISIC-2017 datasets.

Dataset	Backbone/Configuration	Performance Metrics			
		Accuracy (%)	Sensitivity	Specificity	F1-Score
**ISBI-2016**	DenseNet-201				
	Baseline Model	89.50	0.878	0.902	0.886
	Baseline + Enhancement	92.40	0.910	0.931	0.918
	Inception-ResNet v2				
	Baseline Model	88.80	0.869	0.895	0.879
	Baseline + Enhancement	91.60	0.902	0.923	0.908
	NASNet-Mobile				
	Baseline Model	86.90	0.845	0.878	0.855
	Baseline + Enhancement	89.80	0.881	0.904	0.889
	**Proposed Framework**	**96.25**	**0.944**	**0.982**	**0.963**
**ISIC-2017**	DenseNet-201				
	Baseline Model	88.40	0.865	0.893	0.874
	Baseline + Enhancement	91.20	0.898	0.920	0.906
	Inception-ResNet v2				
	Baseline Model	87.70	0.858	0.886	0.868
	Baseline + Enhancement	90.60	0.890	0.914	0.898
	NASNet-Mobile				
	Baseline Model	85.50	0.834	0.866	0.845
	Baseline + Enhancement	88.50	0.869	0.895	0.877
	**Proposed Framework**	**95.85**	**0.956**	**0.960**	**0.956**

**Table 9:** Statistical validation of classification accuracy using one-way ANOVA for the PH^2^ dataset.

Source	SS	df	MS	F-Statistic	Prob > F
Between Classifiers	9.6274	2	4.8137	23.1046	0.0001
Within Classifiers (Error)	2.5001	12	0.2083	–	–
Total	12.1275	14	–	–	–

**Table 10:** Statistical validation of classification accuracy using one-way ANOVA for the ISBI-2016 dataset.

Source	SS	df	MS	F-Statistic	Prob > F
Between Classifiers	15.6484	2	7.8242	55.8885	<0.0001
Within Classifiers (Error)	1.6800	12	0.1400	–	–
Total	17.3284	14	–	–	–

**Table 11:** Statistical validation of classification accuracy using one-way ANOVA for the ISIC-2017 dataset.

Source	SS	df	MS	F-Statistic	Prob > F
Between Classifiers	39.5290	2	19.7645	42.2048	<0.0001
Within Classifiers (Error)	5.6196	12	0.4683	–	–
Total	45.1486	14	–	–	–

**Table 12:** Statistical validation of classification accuracy using one-way ANOVA for the HAM10000 dataset.

Source	SS	df	MS	F-Statistic	Prob > F
Between Classifiers	14.3080	2	7.1540	17.6766	0.0003
Within Classifiers (Error)	4.8566	12	0.4047	–	–
Total	19.1646	14	–	–	–

**Table 13:** Performance comparison of the proposed framework with existing state-of-the-art skin lesion classification methods.

Author (Year)	Problem Type	Dataset	Accuracy
Haque et al. [39] (2026)	Classification	HAM10000	91.15%
Padhy et al. [40] (2025)	-	HAM10000	94.23%
Aruk et al. [24] (2025)	-	HAM10000	94.30%
Hu et al. [41] (2024)	-	ISIC-2017	94.0%
Song et al. [41] (2023)	-	ISIC-2017	95.6%
Alenezi et al. [37] (2023)	-	HAM10000	95.73%
Alhudhaif et al. [42] (2023)	-	HAM10000	95.94%
Benyahia et al. [43] (2022)	-	PH^2^	98.70%
Nakai et al. [44] (2022)	-	ISIC-2017, HAM10000	92.1%, 95.84%
M.K.Hasan et al. [36] (2022)	-	ISBI-2016 & ISIC-2017	92% & 93.1%
Ding et al. [45] (2021)	-	ISIC-2017	92.2 (AUC)%
Calderón et al. [46] (2021)	-	HAM10000	93.21%
Khan et al. [11] (2020)	-	ISBI-2016 & ISIC-2017	94.5% & 93.4%
Hameed et al. [47] (2020)	-	ISBI-2016	96.15%

## Data Availability

The datasets utilized in this study-including HAM10000, ISIC-2017, ISBI-2016, and PH^2^-were retrieved from the Kaggle repository and the official International Skin Imaging Collaboration (ISIC) archives. These datasets are publicly accessible and open to the research community for academic use.
